# Structural and Electrochemical Evolution of Water Hyacinth-Derived Activated Carbon with Gamma Pretreatment for Supercapacitor Applications

**DOI:** 10.3390/ma17133233

**Published:** 2024-07-01

**Authors:** Bordin Weerasuk, Threeraphat Chutimasakul, Nicha Prigyai, Kewalee Nilgumhang, Piriya Kaeopookum, Tanagorn Sangtawesin

**Affiliations:** Thailand Institute of Nuclear Technology (Public Organization), 9/9 Moo 7, Saimoon, Ongkharak, Nakhon Nayok 26120, Thailand; superisaac.017@gmail.com (B.W.); threeraphat.chu@gmail.com (T.C.); nichaprigyai@gmail.com (N.P.); kawalee@tint.or.th (K.N.)

**Keywords:** supercapacitor, gamma irradiation, activated carbons, water hyacinth and clean process

## Abstract

This study introduces a gamma pretreatment of water hyacinth powder for activated carbon (AC) production with improved electrochemical properties for supercapacitor applications. The structural and morphological changes of post-irradiation were meticulously analyzed using scanning electron microscopy (SEM), Raman spectroscopy, Fourier-transform infrared spectroscopy (FT-IR), Brunauer–Emmett–Teller (BET) analysis, and X-ray photoelectron spectroscopy (XPS). The pretreatment significantly modifies the pore structure and reduces the particle size of the resulting activated carbon (WHAC). Nitrogen adsorption-desorption isotherms indicated a substantial increase in micropore volume with escalating doses of gamma irradiation. Electrochemically, the activated carbon produced from pretreated WH at 100 kGy exhibited a marked increase in specific capacitance, reaching 257.82 F g^−1^, a notable improvement over the 95.35 F g^−1^ of its untreated counterpart, while maintaining 99.40% capacitance after 7000 cycles. These findings suggest that gamma-pretreated biomasses are promising precursors for fabricating high-performance supercapacitor electrodes, offering a viable and environmentally friendly alternative for energy storage technology development.

## 1. Introduction

Energy scarcity has emerged as a critical global challenge, exacerbated by the escalating demands of a rapidly growing population and swift economic progression. As a result, the global need for reduced energy consumption has paradoxically led to increased energy demand. To address this, deploying renewable energy sources, such as solar and wind, has been advocated, although this solution necessitates the development of suitable energy storage systems [1,2,3]. Supercapacitors have gained prominence within this context as a component of the energy storage matrix. They are distinguished by their remarkable power density, which exceeds 10 kW kg^−1^, and their ability to charge and discharge at accelerated rates [2,3,4]. In addition to their high energy density and extensive cycle life, which surpasses one million cycles [2,3], supercapacitors are versatile and find applications in various domains, including mobile phones, electric vehicles, power quality management, backup energy supplies, and the integration with renewable energy systems [5,6,7,8].

Supercapacitors, pivotal in advancing energy storage technology, utilize two principal mechanisms for charge accumulation. The first is the electric double-layer capacitor (EDLC), where charge is stored via electrostatic forces at the electrode–electrolyte interface, employing carbon-based materials such as carbon nanotubes [9], carbon nanofibers [10], carbon aerogel [11], graphene [12], and activated carbon (AC) [13]. The second mechanism involves faradaic processes, a characteristic of pseudocapacitors, which store charge through redox reactions on the electrode’s surface, utilizing materials like conductive polymers and transition metal oxide electrodes [14,15,16]. Within this spectrum, AC emerges as a prominent electrode material for EDLCs owing to its high specific surface area, pronounced porosity, excellent electrical conductivity, and ease of heteroatom doping [17,18,19]. Furthermore, AC is non-toxic and offers a sustainable solution to waste management challenges in the agricultural sector and biomass [20], underscoring its significance in energy storage.

The efficacy of activated carbon (AC) in supercapacitors is significantly influenced by its surface area, pore structure, and electrical conductivity; these attributes collectively enhance the charge storage capacity and facilitate charge transfer within the AC framework. Recent advancements have highlighted biomass as a cost-effective carbon precursor derived from diverse biomass sources for AC production. The water hyacinth (Eichhornia crassipes), native to South America and now widespread in regions like Thailand, Indonesia, and China, is a prolific aquatic weed. It can produce up to 14 million progenies annually, covering 1.4 square kilometers, and yield 28,000 tons of fresh biomass [21], often leading to waterway blockages and ecological damage [22,23]. Consequently, the management of water hyacinth has garnered attention due to its chemical composition, which is high in lignocellulose, consisting of 48% hemicellulose, 20% cellulose, and 3.5% lignin, making it a viable candidate for conversion into AC [24]. Morales et al. reported that preparing nitrogen-doped porous carbon from water hyacinth showed a promising AC for oxygen reduction reaction in an alkaline fuel cell [25]. Kurniawan et al. studied the synthesis of carbon microspheres from water hyacinths for supercapacitor electrodes. The carbon microsphere showed suitable specific capacitance and electrochemical stability; the capacitance of AC electrodes exhibited 168.9 F g^−1^ over 1000 cycles [26]. In addition, Saning et al. prepared magnetic carbon composite adsorbents and supercapacitor electrodes from water hyacinth; the electrodes possessed a high specific area of 2545 cm^2^ g^−1^, excellent specific capacitance of 100 F g^−1^ at 1 A g^−1^, and 92% of retention of capacitance after 10,000 cycles [27]. Zheng et al. reported the hierarchical porous from water hyacinth by pre-carbonization and KOH activation for supercapacitor applications and exhibited a high specific area of 2276 m^2^ g^−1^ and 344.9 F g^−1^ of specific capacitance at a current density of 0.5 A g^−1^, with 95% capacitance retention over 10,000 cycles [1]. Recently, Butcha et al. prepared the nitrogen-doped hierarchical activated carbon derived from water hyacinth, with a high nitrogen content of 4.17 wt% and a high specific area of 1353 m^2^ g^−1^ obtained. This material is applied for lithium-ion batteries and supercapacitors, which exhibit a specific capacitance of 93 F g^−1^ at a current density of 0.5 A g^−1^ [23]

In the domain of supercapacitor electrode fabrication, this study investigates the application of gamma irradiation as a pretreatment step for converting water hyacinth into high-quality activated carbon. The gamma irradiation process is lauded for its numerous benefits, including cost-effectiveness, uniform treatment, minimal energy requirements, and scalability potential [28]. During gamma irradiation, water molecules are dissociated into both oxidizing (OH^•^ and H_2_O_2_) and reducing agents (e^−^_q_ and H^•^) [29], effectively deconstructing the lignocellulosic structure into its constituent components: hemicellulose, cellulose, and lignin [30,31,32]. In recent years, the environmental safety, reduced maintenance costs, and energy efficiency of gamma irradiation have made it a preferred method for treating carbon materials [33,34].

Mohd Nor et al. demonstrated the efficacy of gamma irradiation at doses of 5, 15, and 20 kGy in processing activated green monoliths (GMs) derived from oil palm EFB fibers for supercapacitor electrodes [35]. They found that at 5 kGy, they achieved the optimal balance, yielding a specific capacitance of 196 F g^−1^ and providing significant specific power and energy (236 W kg^−1^ and 5.45 Wh kg^−1^, respectively) [35]. Adhamash et al. assessed electrodes fashioned from coconut shell biochar treated with gamma irradiation doses of 50, 100, and 150 kGy. Here, the 100 kGy doses exhibited superior performance, with a specific capacitance of 246.2 F g^−1^, and demonstrated remarkable capacity retention of approximately 97% after 10,000 cycles, coupled with high energy (34.2 Wh kg^−1^) and power densities (0.1 kW kg^−1^) [4]. Recently, Numee et al. reported on the treatment of durian shell biomass powder with gamma irradiation at doses of 10, 30, and 50 kGy, followed by hydrothermal treatment with ZnCl_2_/FeCl_3_ for supercapacitor electrodes. The study highlighted a peak-specific capacitance of 325.20 F g^−1^ at a current density of 1 A g^−1^ achieved at the dose of 30 kGy, with a commendable capacity retention of 94.79% after 10,000 cycles [28]. Concurrently, Benwannmas et al. investigated the carbonization of palm petiole biomass powder via H_2_SO_4_ hydrothermal treatment, KOH-activated pyrolysis, and gamma radiolysis under oxidizing conditions. The treatment with gamma irradiation at 25, 50, and 100 kGy revealed the highest specific capacitance of 309 F g^−1^ at 50 kGy, maintaining excellent capacity through 10,000 charge–discharge cycles [36]. These findings collectively underscore the potential of gamma irradiation treatment in producing supercapacitor electrodes with outstanding specific capacitance and durability.

This research used gamma radiation to pretreat dried water hyacinth leaves before transforming them into high-quality AC for supercapacitor electrodes. This process was conducted in an eco-friendly manner, avoiding the use of toxic chemicals, high temperatures, and chemical residues. We examined the effects of gamma radiation at doses of 50, 100, and 150 kGy on the chemical composition, physical structure, surface morphology, and electrical conductivity of the resulting AC. Additionally, we assessed the performance characteristics of the supercapacitors produced from these materials.

## 2. Materials and Methods

### 2.1. Materials and Instruments

Water hyacinth leaves (RWH) were collected from a river near the Thailand Institute of Nuclear Technology, Nakhon Nayak, Thailand. All chemicals were used without purifying. Potassium hydroxide (KOH, AR grade) was purchased from KEMAUS (Cherrybrook, Australia). Poly (vinylidene fluoride) (PVDF), n-methyl-2-pyrrolidone (NMP) and graphite foil, 0.13 mm (0.005 in)-thick, 99.8%, was obtained from Alfa Aesar (Haverhill, MA, USA). Sulfuric acid (H_2_SO_4_) was purchased from RCI LABSCAN (Bangkok, Thailand). Carbon black was purchased from MTI Corporation (Richmond, CA, USA). Deionized water (DI water) was collected from ultra-pure water ASTM Type I, ThermoScientific (Waltham, MA, USA), with resistivity >18.2 MΩ.

Scanning electron microscopy (SEM, SU5000 Hitachi, Tokyo, Japan) was used to determine morphology. The surface area, pore size and pore volume were analyzed by N_2_ adsorption-desorption (ASAP2460, Micromeritics, Norcross, GA, USA) and surface area was calculated using the Brunauer–Emmett–Teller (BET) model. The Raman spectroscopy (532 nm laser, XploRA PLUS HORIBA, Palaisesu, France) was used to investigate the disordered structure and the graphitic content. Functional groups of all samples were identified by using a Fourier transform infrared spectrometer (FTIR, Bruker Tensor 27, Ettlingen, Germany). Element components and chemical structures were determined by X-ray photoelectron spectroscopy (XPS, AXIS Ultra DLD, Shimadzu, Kyoto, Japan). Cyclic voltammetry (CV), galvanostatic charge/discharge (GCD), and electrochemical impedance spectroscopy (EIS) were conducted by Palmsens4 (Houten, The Netherlands) to determine the electrochemical properties.

### 2.2. Synthesis of Activated Carbon

Dried water hyacinth (WH) powder was sieved with a 200 mesh sieve. Four grams of WH powder were immersed in glass bottles with 200 mL of DI water and sonicated for 60 min. The glass bottles were sealed with parafilm and irradiated with a gamma irradiator (Gamma Chamber 5000, BRIT, Mumbai, India, ^60^Co source and the dose rate of 1.9 kGy hr^−1^) at the dose of 0, 50, 100 and 150 kGy. After that, the mixtures were filtered to separate solid and liquid parts. The solid part was carbonized using TMAX Furnace TL1200 (Fujian, China) at 800 °C for 5 h under N_2_ gas to receive biochar. Then, activation was achieved by mixing biochar with KOH with a ratio of 1:2 by weight and transferred into the oven at 80 °C for 72 h before activation at 800 °C for 5 h under N_2_ gas. The samples were washed with 0.1 M H_2_SO_4_ and DI water and dried at 60 °C for 24 h. The AC synthesized from pre-irradiated WH at 0 (unirradiated), 50, 100, and 150 kGy were named WHAC, WHAC50, WHAC100, and WHAC150, respectively.

### 2.3. Electrochemical Measurement

The electrodes were fabricated from WHAC samples, carbon black, and PVDF (5%*w*) as a ratio of 8:1:1 *w*/*w*. The mixture was dissolved in NMP and sonicated for 30 min. The received slurry was coated on the 2 × 2 cm^2^ graphite foil and dried at 100 °C. This material was used as a working electrode (mass loading is 3–5 mg per electrode) for electrochemical measurement. The CV was tested as three electrode systems at room temperatures with the scan rate range of 10–70 mV s^−1^. Platinum (Pt) wire and Ag/AgCl were used as counter and reference electrodes, respectively. Sulfuric acid (H_2_SO_4_, 2 M) was used as an electrolyte. The EIS was performed at an applied voltage of 10 mV within a frequency of 10 kHz–1 Hz. The GCD was performed at 1–5 A g^−1^ current density.

## 3. Results and Discussion

### 3.1. Materials Characterization

**Figure 1:**
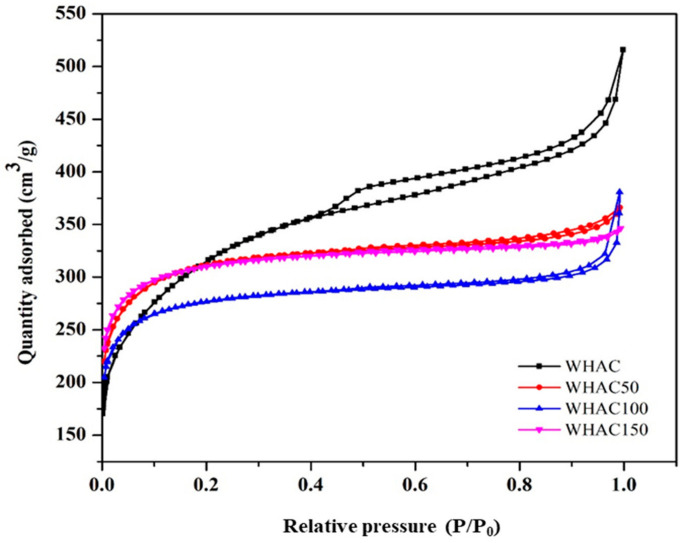
Nitrogen adsorption-desorption isotherm of non-irradiated and gamma-irradiated at different dosages of WHAC.

During gamma pretreatment of WH (cellulose, hemicellulose, and lignin), the ester linkage between lignin and hemicellulose was broken. Also, the cellulose and hemicellulose chains were scissored [37]. All samples’ surface area and porosity were investigated, as shown in Table 1. The data suggests that as the gamma dose increased from 0 to 150 kGy, the BET surface area was slightly lower than WHAC, whereas the micropore area increased. However, 150 kGy exhibited the greatest micropore area of 725.21 m^2^/g, indicating that higher irradiation doses may facilitate the development of microporosity in the carbon structure. The external surface area consistently decreased with increasing irradiation doses. The pore size increased with the dose up to 100 kGy and then slightly decreased to 150 kGy. Notably, the average pore sizes of WHAC, WHAC50, WHAC100, and WHAC150 were larger than those of both SO_4_^2−^ hydrated anions (3.79 Å) and H^+^ hydrated cations (2.80 Å). The WH series’ nitrogen adsorption/desorption isotherm was performed at 77 K, as shown in Figure 1. All samples exhibited a type IV adsorption isotherm with a hysteresis loop associated with capillary condensation [38], limiting uptake over a range of high P/P_0_. It indicated the hysteresis loops of type H4, suggesting narrow slit-like pores with internal voids of irregular shape, broad size distribution, and hollow spheres [39]. Gamma irradiation pretreatment changed adsorption–desorption behavior, indicating the different pore morphology.

**Figure 2:**
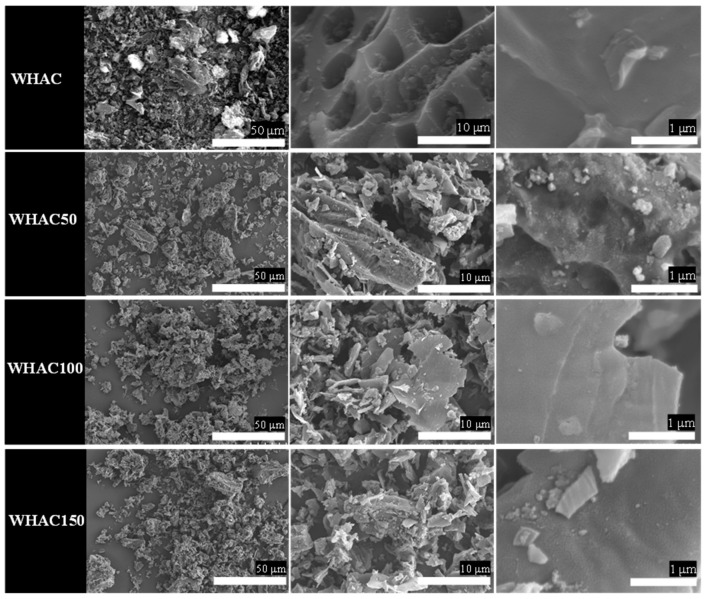
SEM images of WHAC, WHAC50, WHAC100 and WHAC150.

**Figure 3:**
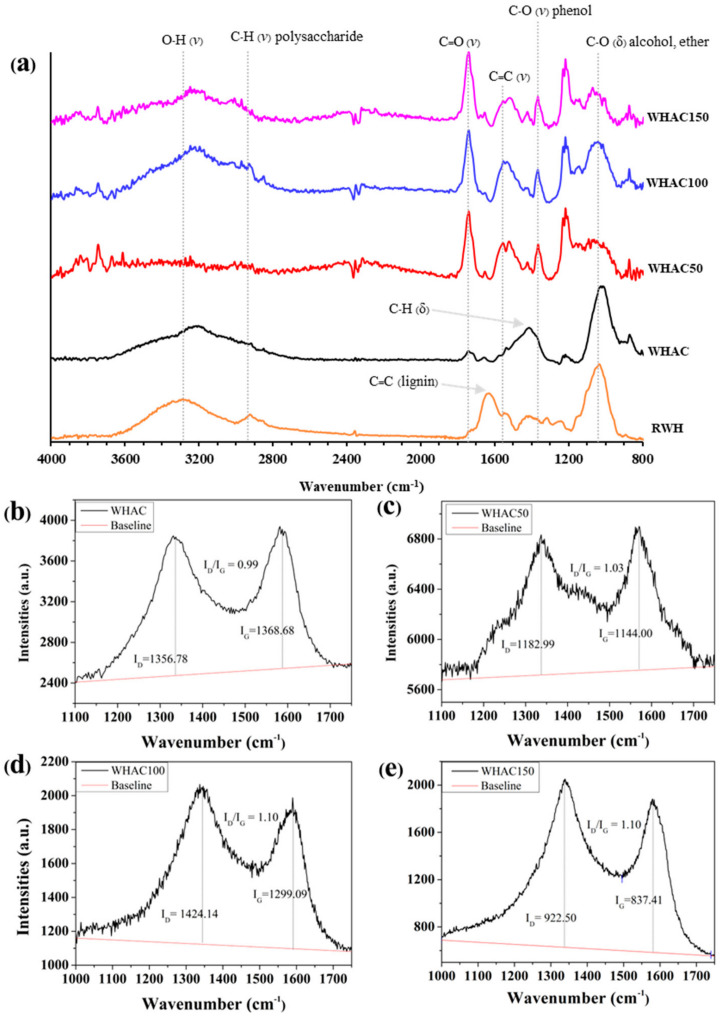
(**a**) FT−IR spectra of WHAC series (δ = symmetric bending vibration and *ν*(s) = stretching to symmetric stretching vibration), Raman spectra of (**b**) WHAC, (**c**) WHAC50, (**d**) WHAC100, and (**e**) WHAC150.

The morphologies of WH samples were further investigated using scanning electron microscopy (SEM), as shown in Figure 2. The original plant cell walls were observed in WHAC. However, rough surfaces with small broken plates were found in WHAC50, WHAC100 and WHAC150. It indicates that gamma pretreatment at 50–150 kGy destroyed the lignocellulose structure and affected AC’s morphology [37]. Moreover, FTIR spectra of raw water hyacinth (RWH) and WHAC series were performed to determine the surface functional groups of materials, as shown in Figure 3a. Notably, the FTIR spectrum of RWH exhibited peaks around 3300, 2910, 1630, and 1030 cm^−1^ according to the OH stretching (alcohol, phenol, and carboxylic acid), C-H stretching of polysaccharide, C=C aromatic stretching [40] and C-O stretching of lignocellulosic structure [41,42], respectively. For the WHAC series, the FTIR spectrum of WHAC exhibits a new weak peak at 1740 cm^−1^ attributed to C=O stretching of the carboxylic group. In addition, peaks at 1400 and 1030 cm^−1^ were observed, corresponding to C-H bending and C-O stretching, respectively. Nonetheless, the FTIR spectra of WHAC50, WHAC100, and WHAC150 showed sharp peaks at 1740 and 1210 cm^−1^, which are related to C=O stretching and C-O stretching of the carboxylic group, which suggests that parts of lignin were decomposed and converted to phenolic and carboxylic acid groups [43]. In addition, the adsorption bands at 1550 and 1350 cm^−1^ were obtained in WHAC50, WHAC100, and WHAC150, assigned to C=C stretching and CH_2_ stretching of aliphatic, respectively [41].

Raman spectra of WHAC samples are shown in Figure 3b. There are two prominent characteristic peaks of carbon materials: the D band corresponds to the doubly resonant disorder-induced carbon structure (1350 cm^−1^), and the G band corresponds to the C=C bond structure in the graphite layer with sp^2^ hybridization (1587 cm^−1^) [44]. The ratio of intensities of the D and G bands (I_D_/I_G_) can indicate the degree of graphitization in materials. A higher I_D_/I_G_ value indicates a higher level of disorder carbon and defects [45,46]. The I_D_/I_G_ of WHAC50 (Figure 3c), WHAC100 (Figure 3d), and WHAC150 (Figure 3d) were 1.03, 1.10, and 1.10, respectively. These I_D_/I_G_ values were slightly higher than untreated WHAC (0.99), as shown in Figure 3b–e. It suggests that the graphitization structure of AC was slightly decreased, and the formation of defects was influenced by neighboring oxygen functional groups [47]. Adding heteroatoms or oxygen functional groups to the electrode surface for supercapacitors boosts hydrophilicity in water-based electrolytes, enhancing charge transfer and increasing capacity [48,49].

**Figure 4:**
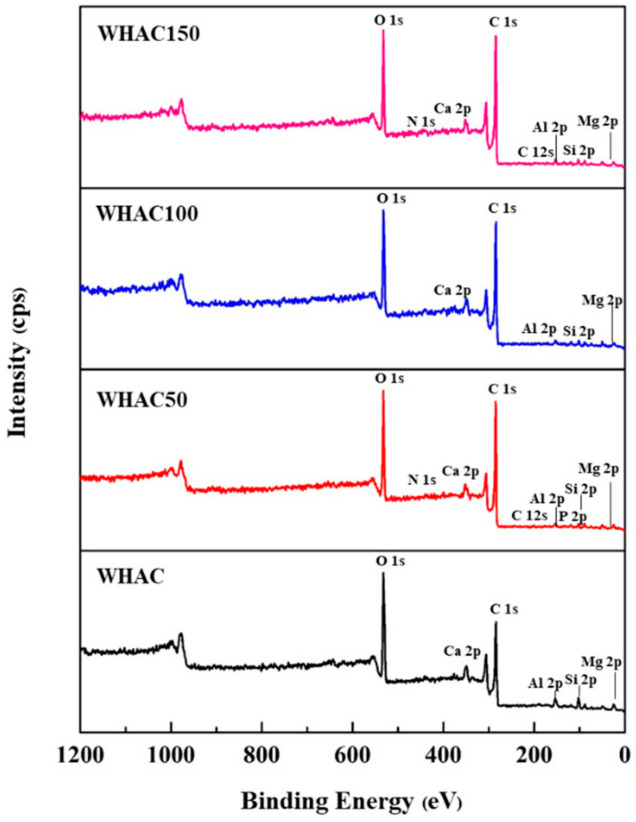
XPS survey spectra of WHAC, WHAC50, WHAC100, and WHAC150.

**Figure 5:**
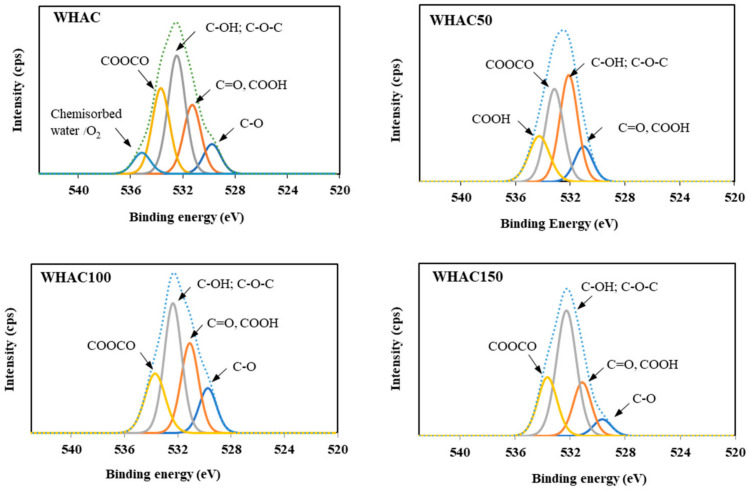
High-resolution XPS spectra of O1s for WHAC, WHAC50, WHAC100, and WHAC150.

The XPS technique was used to determine the elemental compositions of WHAC, WHAC50, WHAC100, and WHAC150. In the XPS survey spectra, carbon and oxygen were the main elements located at the binding energies of 285 and 532 eV (Figure 4), respectively [50,51,52,53]. Also, trace amounts of Ca, Al, Mg, and N were found (Table 2). For the O1s XPS spectra of the WHAC series shown in Figure 5, five main peaks with binding energy at 529, 531, 532, 533, and 535 eV were observed, corresponding to C=O, −COOH/C=O, C-OH/C−O-C, COOCO, and the chemical adsorption of O_2_/H_2_O, respectively [41,54]. WHAC50, WHAC100, and WHAC150 contained carboxylic moieties, consistent with the FTIR results. For WHAC, COOH was found in XPS, but there was no evidence in FTIR. Table S1 WHAC100 contained more C=O/COOH (26.8%) and C-OH/C-O-C (38.9%) than others. The oxygen-containing groups such as hydroxyl, carboxyl, and epoxy groups have oxygen atoms with strong polarity and can interact with water molecules through hydrogen bonding and dipolar forces, resulting in high activity and wettability, which enables ions to access carbon materials pores, thereby enhancing capacitance quickly [53]. Moreover, the high-resolution spectra of C1s were also fitted [52,55,56] and shown in Figure S1 and Table S1. It was found that only WHAC50 contains π-π* at 290 eV for 3.1%.

### 3.2. Electrochemical Performance

**Figure 6:**
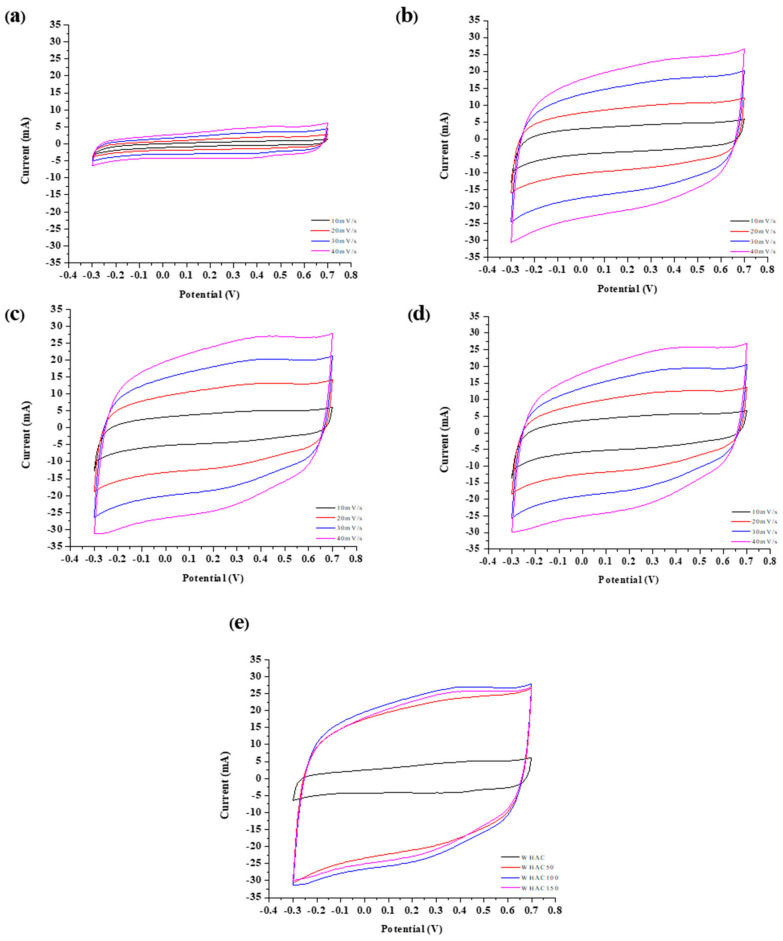
Cyclic voltammograms with applied potential from −0.3 to 0.7 V of (**a**) WHAC, (**b**) WHAC 50, (**c**) WHAC100, (**d**) WHAC150 at different scan rates and (**e**) cyclic voltammograms of all WHAC electrodes with a scan rate of 40 mV s^−1^.

**Figure 7:**
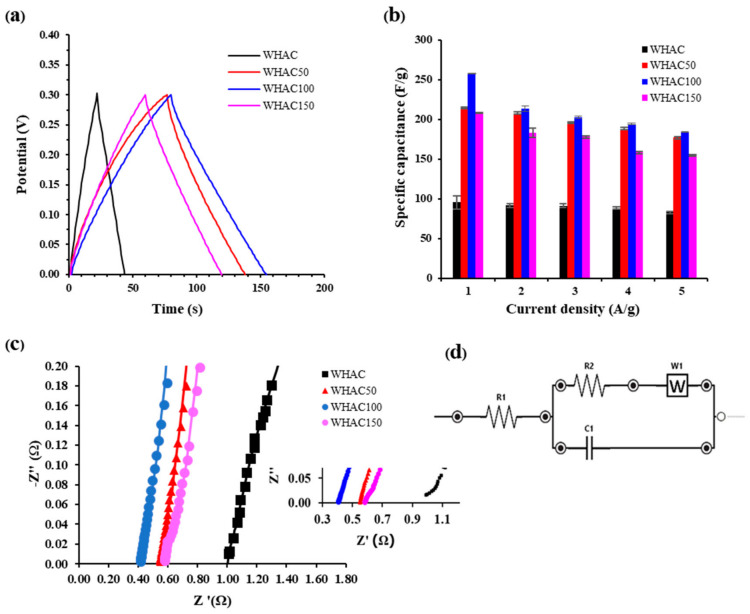
(**a**) Galvanostatic charge–discharge curve of WHAC, WHAC50, WHAC100, and WHAC150. (**b**) The specific capacitance at a different current density 1–5 A g^−1^. (**c**) Nyquist plots of WHAC, WHAC50, WHAC100, and WHAC150 electrodes at 10 mV within a frequency range of 10 kHz–1 Hz. (**d**) Equivalent circuit obtained from curve fitting.

The electrochemical performance was investigated using CV, GCD, and EIS in a three-electrode system. The CV curves at different scan rates (10–70 mV s^−1^) in −0.30 to 0.70 V potential windows of WHAC, WHAC50, WHAC100, and WHAC150 were demonstrated in Figure 6. The voltammograms of WHAC50, WHAC100, and WHAC150 gave a similar shape, as shown in Figure 6b–d. However, the WHAC (Figure 7a) exhibited a different shape, which may result from different porosity and specific surface area of materials [4]. For WHAC50, WHAC100, and WHAC150, the CV curve appeared to have rectangle shapes as increased scan rates, indicating the electrical double-layer capacitance (EDLC) [57,58,59]. In Figure 6e, the area of the CV curves of WHAC50, WHAC100, and WHAC150 are significantly larger than that of WHAC at the same scan rate. This indicates that WHAC tends to have the lowest gravimetric specific capacitance.

The capacitive behavior of the AC-derived supercapacitor electrodes was examined in further detail using galvanostatic charge/discharge (GCD) measurement. The recorded GCD plot of all electrodes at a current density of 1 A g^−1^ is shown in Figure 7a. The GCD curves of WHAC, WHAC50, WHAC100, and WHAC150 electrodes were the perfect triangular shape of an electric double-layer capacitor, but their discharge times differed. The discharge time is relative to the electrode’s discharge duration and capacitance. The specific capacitance (C_p_, F g^−1^) of their electrode systems was evaluated by galvanostatic charge–discharge (GCD) tests according to Equation (1) [36].

(1) $$C_{p}=\frac{I_{m}\times \Delta t}{\Delta V}$$

where C_p_ is the specific capacitance (F g^−1^), I_m_ is the current density (A g^−1^), Δt is the discharging time, and ΔV is the potential drop. The C_p_ at current density 1 A g^−1^ of WHAC, WHAC50, WHAC100, and WHAC150 were 95, 214, 258, and 208 F g^−1^, respectively. Notably, the C_p_ decreases gradually as the current density increases (Figure 7b) due to the limited transportation of electrolyte ions [4,57]. The WHAC100 exhibited the highest capacitance due to the suitable pore morphologies and large surface area. Liu et al. reported that the average pore size of microporous carbons significantly influences their performance in different electrolytes. Specifically, pore sizes smaller than 0.6 nm facilitate enhanced ion penetration and the formation of electric double-layer (EDL) capacitance in KOH. In contrast, larger pore sizes permit the entry of solvated/denaked SO_4_^2^− ions in H_2_SO_4_ [60]. This enhances capacitance through both EDL and pseudocapacitance effects.

Moreover, electrochemical impedance spectroscopy (EIS) was used to evaluate internal resistances. The equivalent series resistance is important in evaluating a capacitor. It depends on various factors, including the intrinsic nature of the electrode material, the pore size distribution of the high-surface area materials used in fabricating the electrodes, and the engineering parameters used in the formulations. Nyquist plots of WHAC, WHAC50, WHAC100, and WHAC150 are shown in Figure 7c. Small semi-circle loops were found at high frequencies of WHAC50, WHAC100, and WHAC150, as shown in the inset of Figure 7c, which identified low resistance at the electrode-electrolyte interface. The slope of Nyquist plots can identify the diffuse layer resistance. WHAC100 has a more significant slope than WHAC, WHAC50, and WHAC150, indicating WHAC100 had the highest conductivity and lower internal resistance. Moreover, the equivalent circuit of Nyquist plots was simulated using ZView3.4 software (Figure 7d), where R_1_ is the internal resistance of the electrodes (R_s_), R_2_ is the polarization resistance of the carbon electrode (R_p_), C_1_ is the capacitor, and W is the Warburg impedance [28,61]. R_s_ is the internal resistance of electrodes, which can be identified at the high-frequency intercept of the plot on the real axis (Z’ axis). Table 3 presents the R_s_ of WHAC50 (0.53 Ω), WHAC100 (0.40 Ω), and WHAC150 (0.62 Ω), compared with WHAC (1.03 Ω). The lower R_s_ for gamma-pretreated WHAC may result from surface oxygen functional groups, as confirmed by FTIR results, providing better wettability of the electrode surface and causing a low interfacial resistance [48,49,53,62].

**Figure 8:**
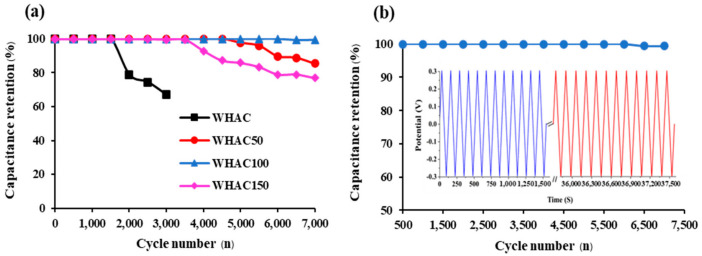
Cyclic stability of (**a**) all WHAC electrodes and (**b**) WHAC100 electrode, which inset figure shows GCD measurement.

The stability of the WHAC, WHAC50, WHAC100, and WHAC150 electrodes was investigated. In Figure 8a, the specific capacitance (C_p_) in the first 1500 cycles was 100% of capacitance retention in all samples. After 3000 cycles, the Cp of WHAC electrode dropped significantly to 67.03%, whereas the C_p_ of WHAC50, WHAC100, and WHAC150 changed slightly. After 7000 cycles, the C_p_ dropped to 85.39%, 99.45%, and 76.87%, respectively. This result suggests that WHAC100 showed better cycle stabilities than other samples, which remained at 100% retention over 6500 cycles, then slightly decreased to 99.40%, and stayed consistent at this value beyond 7000 cycles, as shown in Figure 8b.

**Figure 9:**
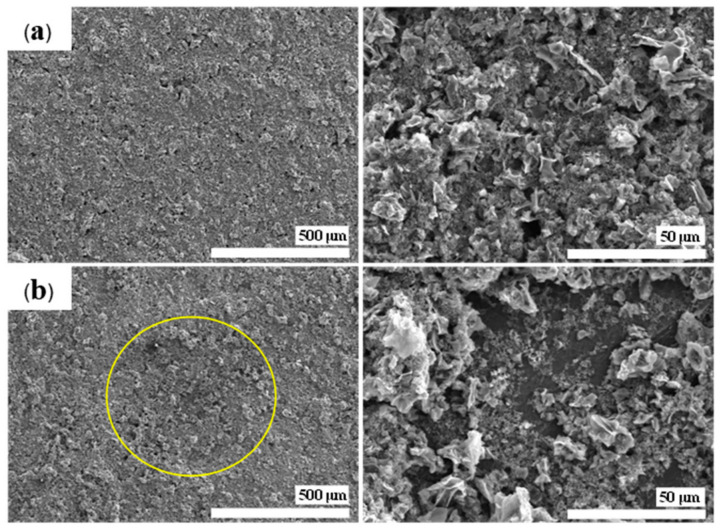
SEM images of WH100 electrode, (**a**) before and (**b**) after the charge–discharge cycle stability test.

Figure 9 shows the morphological examination of the WHAC100 electrode, conducted before and after the cycle stability test for 7000 cycles. The SEM images indicate that a portion of the material’s surface experienced slight degradation post-testing, in contrast to the pristine state of the WHAC100 electrode. These findings confirmed that the WHAC100 electrode retained superior performance in supercapacitor applications after 7000 cycles. Therefore, the gamma pretreatment method affected the electrochemical properties of activated carbon derived from water hyacinth, including their specific capacitance, capacitance retention, and cycling stability, making it a promising approach for advancing supercapacitor technology.

Compared to other studies in Table 4, Jujube fruit-derived materials exhibited the highest specific capacitance of 460 F g^−1^. In contrast, materials derived from durian shells showed the lowest, with a capacitance of 178 F g^−1^. The WHAC material demonstrated a commendable specific capacitance of 258 F g^−1^. Furthermore, electrodes fabricated from sugarcane bagasse, when paired with a 1 M H_2_SO_4_ electrolyte, achieved a specific capacitance of 298 F g^−1^, alongside a cycle stability retention capacitance of 94.5% after 5000 cycles. Notably, the WHAC electrode, utilizing a similar electrolyte, achieved a comparable specific capacitance but exhibited superior cycle stability, with a retention capacitance of 99.4% after 7000 cycles. This underscores the efficacy of the WHAC electrode in maintaining electrochemical performance over extended cycling. Consequently, this study presents a viable alternative approach for fabricating supercapacitor electrodes with excellent electrochemical performance. This comparison highlights the potential of the WHAC material in supercapacitor applications, particularly in terms of its cycle stability and overall electrochemical efficiency.

## 4. Conclusions

High-quality activated carbons derived from water hyacinth biomass were successfully synthesized using gamma pretreatment at 50, 100, and 150 kGy for supercapacitor electrode applications. The gamma pretreatment was found to significantly influence the chemical structure, surface area, and porosity of the water hyacinth, enhancing its electrochemical properties. WHAC100 (pretreated at 100 kGy) exhibited the highest specific capacitance of 257.82 F g^−1^ and maintained approximately 99% capacity retention after 7000 cycles. In contrast, the untreated WHAC showed a specific capacitance of 95.35 F g^−1^. WHAC100 demonstrated superior characteristics, including the lowest internal resistivity (0.40 Ω), which is about half that of untreated WHAC, a larger pore size (7.23 nm), and the presence of oxygen functional groups. This indicates that pretreatment at 100 kGy developed a suitable pore morphology and chemical properties, considerably improving the specific capacitance. This work demonstrates that gamma pretreatment is an eco-friendly, effective method for producing biomass-derived activated carbon for electrical double-layer capacitor applications in energy storage.

## Figures and Tables

**Table 1:** The BET and pore structural parameters of the WH series.

Samples	BET Surface Area (m^2^/g) ^a^	Micropore Area (m^2^/g) ^b^	Average Pore Size (nm) ^c^
WHAC	1038.70	251.39	3.99
WHAC50	966.45	642.10	3.43
WHAC100	856.61	643.93	7.23
WHAC150	957.34	725.21	3.47

^a^ Brunauer–Emmett–Teller (BET) method. ^b^ Determined by the t-plot method at P/P_0_ = 0.95. ^c^ BJH desorption.

**Table 2:** Elemental analysis of activated carbon derived from water hyacinth with different gamma doses by X-ray photoelectron spectroscopy (XPS).

Samples	Atomic Concentration (%)								
	C_1s_	O_1s_	Ca_2p_	Si_2p_	Al_2p_	Mg_2p_	N_1s_	C_l2p_	P_2p_
WHAC	60.6	26.2	2.7	5.7	1.6	3.2	-	-	-
WHAC50	70.3	21.5	1.4	2.1	1.4	1.9	0.8	0.2	0.4
WHAC100	72.5	21.6	1.5	1.3	1.0	2.2	-	-	-
WHAC150	74.1	18.1	1.6	2.0	0.9	2.1	0.7	0.5	-

**Table 3:** Resistance of electrode (R_s_).

Sample	R_s_ (Ω)
WHAC	1.03
WHAC50	0.53
WHAC100	0.40
WHAC150	0.62

**Table 4:** Comparison of various biowaste-derived activated carbons as electrode material.

Biowaste Source	Chemical Activation	C_p_ (F g^−1^) at 1 A g^−1^	Electrolyte	Cycle Stability	Ref.
Onion husk	K_2_CO_3_	188	1M TEABF_4_	92.5% (2000 cycles)	[63]
Peanut shell	ZnCl_2_	340	1M H_2_SO_4_	95.3% (10,000 cycles)	[64]
Watermelon rind	KOH	333	6M KOH	96.8% (10,000 cycles)	[65]
Tea leaves	KOH	330	2M KOH	92.0% (2000 cycles)	[20]
Sugarcane bagasse	KOH	298	1M H_2_SO_4_	94.5% (5000 cycles)	[66]
Jujube fruit	NaOH	460	6M KOH	92.2% (130,000 cycles)	[67]
Durian shell	KOH	178	1M KOH	99.0% (4000 cycles)	[46]
Palm petiole	KOH	309	1M KOH	94.0% (10,000 cycles)	[36]
Water hyacinth leaves	KOH	257	2M H_2_SO_4_	99.4% (7000 cycles)	This work

## Data Availability

The original contributions presented in the study are included in the article/Supplementary Materials, further inquiries can be directed to the corresponding author/s.

## Supplementary Materials

The following supporting information can be downloaded at: https://www.mdpi.com/article/10.3390/ma17133233/s1, Table S1: The summarized C 1s and O 1s XPS spectra result for WHAC series.; Figure S1: High-resolution XPS spectra of C1s for WHAC, WHAC50, WHAC100, and WHAC150.
