# Supplementary material for: Structural and Electrochemical Evolution of Water Hyacinth-Derived Activated Carbon with Gamma Pretreatment for Supercapacitor Applications
Source: Materials (Basel). 2024 Jul 1;17(13):3233. doi: 10.3390/ma17133233 (PMC11242469; doi:10.3390/ma17133233)
Supplement: Supplementary file 1 [file materials-17-03233-s001.zip › materials-3005630-supplementary.pdf]

Supporting information

Structural and Electrochemical Evolution of Water Hyacinth-Derived Activated Carbon with Gamma Irradiation Pretreatment for Supercapacitor Applications

Bordin Weerasuk[1], Threeraphat Chutimasakul[1], Thipthanya Duangmanee[1], Nicha Prigyai[1], Kawalee Nilgumhang[1] and Tanagorn Sangtawesin[1*]

[1]Thailand Institute of Nuclear Technology (Public Organization), 9/9 Moo 7, Saimoon, Ongkharak, Nakhon Nayok 26120, Thailand

[*]Corresponding Author e-mail address: tanagorn@tint.or.th

Table S1. The summarized C 1s and O 1s XPS spectra result for WHAC series.

Samples	Spectrum	Assignment	Binding Energy(eV)	%Component
WHAC	C 1s	Carbide	282.0	4.9
		Carbide	282.8	6.2
		C-Si	283.5	5.3
		C=C (sp^2)	284.3	7.7
		C-C (sp^3)	285.1	44.5
		C-O	286.0	18.1
		C=O	287.2	8.7
		carbonates	288.5	4.6
	O 1s	C-O	529.8	9.3
		C=O, COOH	531.3	21.3
		C-OH; C-O-C	532.5	36.4
		COOCO	533.7	26.3
		Chemisorbed water/O_2	532.2	6.6
WHAC50	C 1s	C-Si	283.2	1.1
		C=C (sp^2)	284.1	4.6
		C-C (sp^3)	285.0	60.9
		C-O	286.0	10.4
		C-O	286.9	10.1
		C=O	288.0	5.5
		Carbonate	289.2	4.3
		π-π* (sp^2)	290.5	3.1
	O 1s	C=O, COOH	531.0	12.2

		C-OH; C-O-C	532.5	37.2
		COOCO	533.2	32.2
		COOH	534.3	18.4
WHAC100	C 1s	Carbide	282.2	4.8
		C-Si	282.9	7.1
		C=C (sp^2)ǂ	283.8	4.6
		C-C (sp^3)	284.9	56.8
		C-O	286.0	11.1
		C=O	286.9	9.1
		COOH/COOR	288.1	3.5
		Carbonate	289.3	3.0
	O 1s	C-O	529.8	13.5
		C=O, COOH	531.1	26.8
		C-OH, C-O-C	532.4	38.9
		COOCO	533.7	20.8
WHAC150	C 1s	Carbide	282.3	4.6
		C-Si	283.1	3.3
		C=C (sp^2)	284.2	5.5
		C-C (sp^3)	285.0	60.6
		C-O	286.1	10.9
		C=O	287.1	8.0
		COOH/COOR	288.2	3.4
		Carbonate	289.3	3.8
	O 1s	C-O	529.7	6.2
		C=O, COOH	531.1	19.7
		C-OH, C-O-Cǂ	532.3	51.1
		COOCO	533.6	23.0

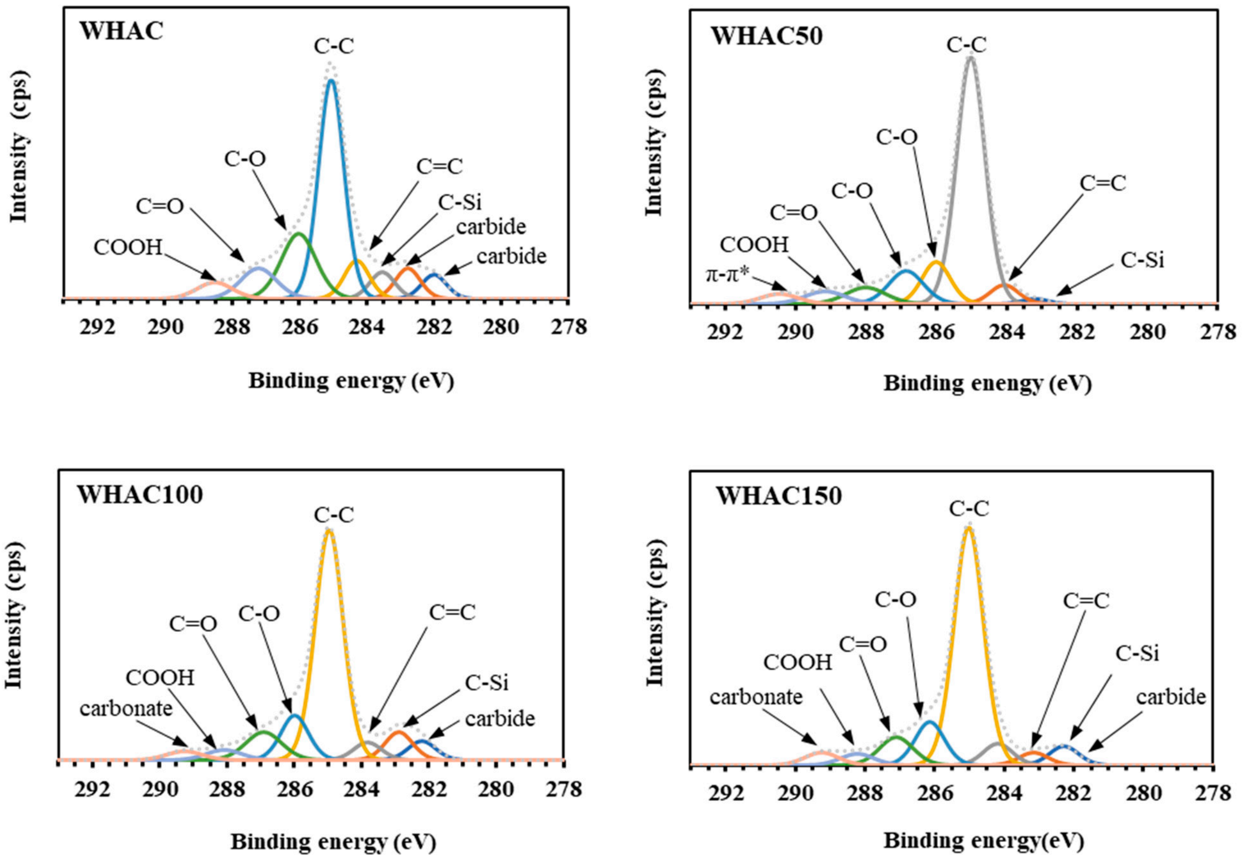

Figure S1. High-resolution XPS spectra of C1s for WHAC, WHAC50, WHAC100, and WHAC150.
